# Supplementary material for: Optimizing experimental design in neutron reflectometry
Source: J Appl Crystallogr. 2022 Jun 23;55(Pt 4):769–81. doi: 10.1107/S1600576722003831 (PMC9348865; doi:10.1107/S1600576722003831)
Supplement: Supplementary file 1 [file j-55-00769-sup1.pdf]

JOURNAL OF APPLIED CRYSTALLOGRAPHY

Volume 55 (2022)

Supporting information for article:

Optimizing experimental design in neutron reflectometry

James H. Durant, Lucas Wilkins and Joshaniel F. K. Cooper

Optimising experimental design in neutron reflectometry – Supporting Information

James H. Durant[1], Lucas Wilkins[2], and Joshaniel F. K. Cooper[1]

[1]ISIS Neutron and Muon source, Rutherford Appleton Laboratory, Harwell Campus, OX11 0QX
[2]School of Life Sciences, University of Sussex, Falmer, Brighton, BN1 9QG

A Experimental Design Optimisation

A.1 Importance Scaling

We quantify parameter importance by choosing a suitable range for each parameter based on physical constraints. These ranges are then mapped to $[0, 1]$. In this way, all parameters are put into the same, non-informational, importance units: a measure of information per importance. Such a linear mapping is analogous to a uniform prior in Bayesian statistics.

To transform the parameters of the Fisher information (FI) matrix, $\mathbf{g}^\xi$, we calculate $\mathbf{J}^\mathbf{T}\mathbf{g}^\xi\mathbf{J}$, where $\mathbf{J}$ is the Jacobian for the transform. Since the importance mapping relationship is linear, the transform will be of the form $y_i = m_i x_i + c_i$ and $\mathbf{J}$ will be a diagonal matrix with entries m_i, i.e., each entry of the FI matrix, $\mathbf{g}^\xi_{i,j}$, is scaled by m_i times m_j. For example, if parameter i is 10 times more important than parameter j, then the m_i will be 10 times m_j. Say the specified interval for a parameter, x, is $[a, b]$, then the linear transform to y, with interval $[0, 1]$, is of the form

$$y = (x - a)/(b - a) = x/(b - a) - a/(b - a)$$

In the form $y = mx + c$, we have $m = 1/(b - a)$ and $c = a/(b - a)$, and hence the required values for the coordinate transform.

A.2 Maximin Optimisation

The presence of information content in individual parameters may hide low information content in a combination of parameters. Our approach chooses the (linear) combination of values that (locally) has the least information content and maximises it. Figure 1 (right) shows an ellipse representing the FI, and how the ellipse corresponds to the amount of information in each parameter. The eigenvector of the FI matrix with minimum eigenvalue corresponds to the minor (short) axis of the ellipse and will be the linear combination of values with the least information.

The details of the mathematics describing the information quantities in figure 1 are as follows: consider two pairs of nearby parameters, one represented by a point in 2D parameter space p and

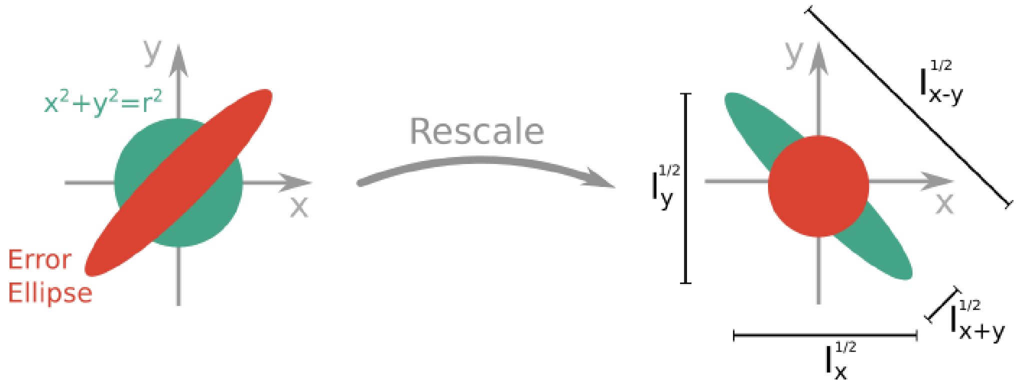

Figure 1: Shown is an error ellipse with a large relative error in a combination of parameters (the $x + y$ combination). Although there is information in both x and y, there is relatively little in the $x + y$ direction. *Details:* If we linearly transform the space on the left so that the error ellipse becomes the unit circle, the unit circle will be transformed into an inverted form of the error ellipse with sizes corresponding to the information content.

another by $p + \Delta p$, where the magnitude of Δp is small and equal to r. The information divergence between these points is given by

$$D\left(p \,\|\, p + \Delta p\right) = \frac{1}{2} \sum_{ij} g_{ij} \Delta p_i \Delta p_j + O\left(\|\Delta p\|^3\right)$$

If Δp is in the direction of the x-axis we can get an approximation which we will call I_x

$$D\left(p \,\|\, p + \Delta p\right) \approx \frac{1}{2} r^2 g_{xx} = I_x$$

Similarly, in the y direction we have $I_y = r^2 g_{yy}/2$, and in the 45° directions, $x + y$ and $x - y$, we have

$$I_{x+y} = \frac{1}{4} r^2 (g_{xx} + g_{yy}) + \frac{1}{2} r^2 g_{xy} = \frac{1}{2}\left(I_x + I_y + r^2 g_{xy}\right)$$

$$I_{x-y} = \frac{1}{2}\left(I_x + I_y - r^2 g_{xy}\right)$$

From this we can see that the total information is the same, whether we use a basis of x and y, or of $x + y$ and $x - y$:

$$I_x + I_y = I_{x+y} + I_{x-y}$$

But we also see that, if the off-diagonal entry of g (i.e., g_{xy}) is either very positive or very negative, the information about $x + y$ or $x - y$ might be very small.

A.3 Parameterisations and Fitting

Sample	Model Parameter	Fitted Value
DMPC Bilayer	Si/SiO_2 Roughness	2.0Å
	SiO_2 Thickness	14.7Å
	SiO_2/DMPC Roughness	2.0Å
	SiO_2 Hydration	24.5%
	DMPC Area Per Molecule	49.9Å^2
	Bilayer Roughness	6.6Å
	Bilayer Hydration	7.4%
	Headgroup Bound Waters	3.59
DPPC/RaLPS Bilayer	Si/SiO_2 Roughness	5.5Å
	SiO_2 Thickness	13.4Å
	SiO_2/Bilayer Roughness	3.2Å
	SiO_2 Hydration	3.8%
	Inner Headgroup Thickness	9.00Å
	Inner Headgroup Hydration	39.0%
	Bilayer Roughness	4.0Å
	Inner Tailgroup Thickness	16.7Å
	Outer Tailgroup Thickness	14.9Å
	Tailgroup Hydration	0.9%
	Core Thickness	28.7Å
	Core Hydration	26.0%
	Asymmetry Value	0.95
DPPG Monolayer	Air/Tailgroup Roughness	5.0Å
	Tailgroup/Headgroup Roughness	2.0Å
	Headgroup/Water Roughness	3.5Å
	Lipid Area Per Molecule	54.1Å^2
	Headgroup Bound Waters	6.69

Table 1: Model parameters and associated fitted values for the DMPC bilayer, DPPC/RaLPS bilayer and DPPG monolayer models.

A.3.1 Lipid Bilayers

Details of the model parameterisation for the 1,2-dimyristoyl-*sn*-glycero-3-phosphocholine (DMPC) bilayer model can be found in our previous work and will not be repeated here. Table 1 summarises the fitted parameters of the model. For the 1,2-dipalmitoyl-*sn*-glycero-3-phosphocholine (DPPC)/Ra lipopolysaccharide (LPS) bilayer model, the level of instrument background for the D_2O (6.14×10^{-6}Å^{-2}), silicon-matched water (2.07×10^{-6}Å^{-2}) and H_2O (-0.56×10^{-6}Å^{-2}) data were 4.6×10^{-6}, 8.6×10^{-6} and 8.7×10^{-6} respectively. Each experimental data set was fitted with an instrument resolution function of constant 4% dQ/Q and an experimental scale factor of 0.8. Table 1 summarises the fitted parameters of the model. The DPPC/RaLPS bilayer model was defined using a slab representation: silicon, silicon oxide, inner headgroup, inner tailgroup, outer tailgroup, LPS core region and finally the bulk water solution of given scattering length density (SLD), ρ_{water}.

The model was defined with three roughness parameters: the silicon/silicon oxide and silicon oxide/bilayer interfacial roughnesses and a bilayer roughness that was shared between the other interfaces (inner headgroup/inner tailgroup, inner tailgroup/outer tailgroup, outer tailgroup/LPS core and LPS core/solution). The silicon substrate layer was defined using the known SLD of silicon ($2.07 \times 10^{-6}\text{Å}^{-2}$). The silicon oxide and inner headgroup layers were defined using their known SLDs ($3.41 \times 10^{-6}\text{Å}^{-2}$ and $1.98 \times 10^{-6}\text{Å}^{-2}$ respectively), with the thickness and hydration of each layer set as parameters. The inner and outer tailgroup layers were defined using separate thickness parameters but a shared hydration parameter. The SLDs for the two layers, $\rho_{\text{inner}_{\text{TG}}}$ and $\rho_{\text{outer}_{\text{TG}}}$, were defined using an asymmetry parameter, α, and the known SLDs of the DPPC and LPS tailgroups ($7.45 \times 10^{-6}\text{Å}^{-2}$ and $-0.37 \times 10^{-6}\text{Å}^{-2}$ respectively).

$$\rho_{\text{inner}_{\text{TG}}} = \alpha\rho_{\text{DPPC}_{\text{TG}}} + (1-\alpha)\rho_{\text{LPS}_{\text{TG}}}$$

$$\rho_{\text{outer}_{\text{TG}}} = (1-\alpha)\rho_{\text{DPPC}_{\text{TG}}} + \alpha\rho_{\text{LPS}_{\text{TG}}}$$

The LPS core layer thickness and hydration were set as parameters, but the SLD was defined using the mole fraction of D_2O from the bulk water SLD, denoted here as x, and the known SLDs of the LPS core in D_2O, $\rho_{\text{core}_{D_2O}}$, and H_2O, $\rho_{\text{core}_{H_2O}}$ ($4.20 \times 10^{-6}\text{Å}^{-2}$ and $2.01 \times 10^{-6}\text{Å}^{-2}$ respectively).

$$x = \frac{\rho_{\text{water}} - \rho_{H_2O}}{\rho_{D_2O} - \rho_{H_2O}}$$

$$\rho_{\text{core}} = x\rho_{\text{core}_{D_2O}} + (1-x)\rho_{\text{core}_{H_2O}}$$

A.3.2 Kinetics

The 1,2-dipalmitoyl-sn-*glycero*-3-phospho-(1-rac-glycerol) (DPPG) monolayer model was defined using a slab representation: air, monolayer tailgroup (either hydrogenated or deuterated), monolayer headgroup, and finally the bulk water solution of given SLD, ρ_{water}; table 1 summarises the parameters of the model. All model interfacial roughnesses (air/tailgroup, tailgroup/headgroup and headgroup/water) were parameterised. The tailgroup (both hydrogenated and deuterated) and headgroup thicknesses were defined using a shared lipid area per molecule (APM) parameter and the equation $d = \text{V}/\text{A}$, where d is the layer thickness, V is the layer volume and A is the lipid APM.

The tailgroup and headgroup volumes were calculated from the volumes of their constituent components, as summarised in table 2. For the tailgroup volume, V_{TG}, this was relatively straightforward

$$V_{\text{TG}} = 28V_{\text{CH}_2} + 2V_{\text{CH}_3}$$

but for the headgroups, we needed to account for the hydrating water molecules. We did this by first multiplying the known water volume by the headgroup bound waters parameter to obtain the extra water volume in the headgroups, V_{bound}, and then added this to the individual fragment volumes

$$V_{\text{HG}} = V_{\text{PO}_4} + 2V_{\text{C}_3\text{H}_5} + 2V_{\text{CO}_2} + V_{\text{bound}}$$

We calculated the hydrogenated and deuterated monolayer tailgroup SLDs using the previously calculated tailgroup volume and equation $\rho = \Sigma b/\text{V}$, where ρ is the layer SLD, Σb is the neutron scattering length (SL) sum for the layer and V is the layer volume. The SL sums of the hydrogenated

and deuterated tailgroups, Σb_{hTG} and Σb_{dTG} respectively, were calculated from the total SL of each constituent fragment. The SLs of the individual elements of the fragments are given in table 3.

$$\Sigma b_{\mathrm{hTG}} = 28\Sigma b_{\mathrm{CH_2}} + 2\Sigma b_{\mathrm{CH_3}}$$
$$\Sigma b_{\mathrm{dTG}} = 28\Sigma b_{\mathrm{CD_3}} + 2\Sigma b_{\mathrm{CD_3}}$$

Like the tailgroups, the headgroup SLD was determined using the previously calculated headgroup volume, the SL sums of the constituent fragments, and equation $\rho = \Sigma b/\mathrm{V}$. However, as with the headgroup volume calculation, we needed to account for the hydrating water molecules. We did this by first calculating the mole fraction of D_2O from the bulk water SLD, x, to get the average SL sum per water molecule.

$$x = \frac{\rho_{\mathrm{water}} - \rho_{\mathrm{H_2O}}}{\rho_{\mathrm{D_2O}} - \rho_{\mathrm{H_2O}}}$$
$$\Sigma b_{\mathrm{water}} = x\Sigma b_{\mathrm{D_2O}} + (1-x)\Sigma b_{\mathrm{H_2O}}$$

By multiplying this value by the headgroup bound waters parameter, we were able to obtain the SL sum of the hydrating water molecules, $\Sigma b_{\mathrm{bound}}$, from which we could calculate the headgroup SL sum.

$$\Sigma b_{\mathrm{HG}} = \Sigma b_{\mathrm{PO_4}} + 2\Sigma b_{\mathrm{C_3H_5}} + 2\Sigma b_{\mathrm{CO_2}} + \Sigma b_{\mathrm{bound}}$$

Fragment	Volume (Å^3)
CH_2	28.1
CH_3	26.4
CO_2	39.0
C_3H_5	68.8
PO_4	53.7
Water	30.4

Table 2: Volumes of the DPPG monolayer tailgroup and headgroup fragments.

Component	Scattering Length (10^{-4}Å)
Carbon	0.6646
Oxygen	0.5843
Hydrogen	-0.3739
Phosphorus	0.5130
Deuterium	0.6671

Table 3: Neutron scattering lengths for the components of the tailgroup and headgroup fragments.

A.3.3 Magnetism

The experimental scale factor, level of instrument background and instrument resolution function used to fit the data were 1.025, 4×10^{-7} and constant 2.8% dQ/Q respectively. The model was defined using a slab representation consisting of air, platinum, yttrium oxide, yttrium iron garnet (YIG) and yttrium aluminium garnet (YAG); table 4 summarises the fitted parameters.

Layer	SLD (10^{-6}Å^{-2})	Thickness (Å)	Roughness (Å)	Magnetic SLD (10^{-6}Å^{-2})
Air	0.00	-	-	-
Platinum	5.65	21.1	8.2	0.00
Yttrium Oxide	4.68	19.7	2.0	-
YIG	5.84	713.8	13.6	0.35
YAG	5.30	-	30.0	-

Table 4: Fitted SLD, thickness, roughness and magnetic SLD for each layer of the magnetic sample.
